# Swept-source optical coherence tomography in ocular surface diseases: anterior segment analysis repeatability and its limits

**DOI:** 10.3389/fmed.2024.1385294

**Published:** 2024-08-02

**Authors:** Alberto Recchioni, Abinaya Priya Venkataraman, Saaeha Rauz, Alberto Domínguez-Vicent

**Affiliations:** ^1^Academic Unit of Ophthalmology, Institute of Inflammation and Aging, University of Birmingham, Birmingham, United Kingdom; ^2^Birmingham and Midland Eye Center, Sandwell and West Birmingham NHS Trust, Birmingham, United Kingdom; ^3^Optometry and Vision Sciences Group, School of Life and Health Sciences, Aston University, Birmingham, United Kingdom; ^4^Division of Eye and Vision, Department of Clinical Neuroscience, Karolinska Institutet, Solna, Sweden

**Keywords:** ocular surface disorders, repeatability, anterior segment OCT, Corneal topography, anterior segment parameters

## Abstract

**Purpose:**

This study aims to evaluate the repeatability of anterior segment optical coherence tomography (AS-OCT) in diverse ocular surface disorder (OSD) cohorts, exploring various anterior segment parameters and their accuracy in different disease groups.

**Methods:**

A total of 239 participants across six distinct OSD groups and healthy controls underwent nonmydriatic AS-OCT imaging using the Tomey CASIA 2 device. Anterior segment parameters including anterior chamber depth, width, angle metrics, corneal thickness, keratometry, lens vault, and others were meticulously assessed. Statistical analyses determined repeatability limits and coefficients of variation for each parameter within the different OSD cohorts.

**Results:**

Repeatability for anterior chamber and corneal parameters remained consistent across all OSD groups, indicating minimal impact of ocular surface disease on accuracy. The coefficient of variation (CoV) for the trabecular iris-space area was about 20% for all cohorts. Ocular surface inflammation emerged as a key factor in dry eye, affecting immune-mediated and non-immune conditions alongside age-related ocular surface changes. While anterior chamber depth measurements showed variations, particularly in immune (CoV = 2.5%) and non-immune (CoV = 3.8%) OSD groups, parameters like anterior chamber width and angle to angle showed similar values among the cohorts. Keratometry measures remained stable despite OSD (CoV lower than 1%).

**Conclusion:**

The Tomey CASIA 2 demonstrated reliable repeatability for measuring anterior segment parameters in diverse OSD cohorts. Despite challenges posed by dry eye conditions, this technology holds promise in assessing OSD, suggesting potential clinical protocols similar to those in healthy controls.

## Introduction

The ocular surface plays a pivotal role in maintaining ocular health by providing a barrier to the external environment. As previously defined by Gipson (1), the ocular surface is seen a system composed of “*the surface and glandular epithelia of the cornea, conjunctiva, lacrimal gland, accessory lacrimal glands, and meibomian gland, and their apical (tears) and basal (connective tissue) matrices, the eyelashes with their associated glands of Moll and Zeis, those components of the eyelids responsible for the blink, and the nasolacrimal duct.*”

The failure of mechanisms responsible for maintaining a healthy ocular surface is underpinned by a group of disorders of diverse pathogenesis leading to ocular surface disease (OSD) (2). A feature of complex OSD is dry eye disease driven by inflammation in many autoimmune-driven conditions including Sjögren’s Syndrome, Ocular Mucous Membrane Pemphigoid, Stevens-Johnson Syndrome (3). Additionally, non-immune conditions such as meibomian glands disease, corneal transplantation, chemical injury or trauma can also lead to ocular surface disease. Dry eye symptoms and therapy have major impact on patients’ quality of life leading to anxiety and depression (4–6).

Ocular surface disorders can be described within five clinical domains (tear film; eyelids, lid margins and meibomian glands (MG); conjunctiva and fornices; cornea; anterior chamber and sclera) across defining descriptors of activity, damage and a relevant clinical assessment /investigation toolkit (3). The latter includes tear film osmolarity, MG secretion quality, fluorescein and lissamine green staining, and a number of visual function tests (in-vivo confocal microscopy (IVCM), anterior segment optical coherence tomography (AS-OCT), ocular surface analyser (OSA), ultrasound biomicroscopy (UBM), Spectroscopy, topography, photography, angiography).

Despite being commonly used in clinic, the impact of objective metrics that consider ocular vital dyes, flashing lights (white and blue cobalt illumination) and tear break-up time tests (e.g., tear evaporation stress) are still posed into debate (7). Instilling vegetable-based dye (e.g., fluorescein) into an altered ocular surface in OSD might lead the clinician to false conclusions because it might induce discomfort and reflex tearing; in fact, an uncontrolled amount of instilled fluorescein can be potentially seen as patches of corneal dryness instead of oversaturation of the stained epithelial cells (8).

Recently, a plethora of new anterior segment visual function medical devices have entered the clinical arena that are non-contact and do not require the use of vital dyes to enhance visualization of pathology or delivering objective metrics that quantify disease outcomes. These include interferometry, infra-red meibography, corneal epithelial thickness, non-invasive ocular surface analysis and devices such as AST-OCT. The evaluation of the anterior segment parameters, such as corneal thickness and curvature, anterior chamber depth (ACD), width, and drainage angle are performed during new and follow-up visits in a range of subspecialty clinics (corneal, cataract, glaucoma) (9–11). It is important to assess how reliable these anterior segment measurements are in the presence of OSD, as the altered ocular surface epithelium, goblet cell loss, anormal tear film and keratinization may influence the accuracy of these measurements. Previous studies have shown that the repeatability of the corneal and anterior chamber parameters is related to the qualitative and quantitative tear film metrics (12, 13). Repeatability of the newer devices in the context of ocular surface dryness is unclear. Scan resolution, acquisition time and the internal algorithm are not fully validated for patients with an ocular surface condition. In this study, we have evaluated the repeatability of a newer swept-source AS-OCT (ss-AS-OCT) Casia2 (Tomey, Japan) able to obtain non-invasive high-resolution cross-section of biological structures using low-coherence light. By considering ss-AS-OCT technology in a range of OSD versus healthy cohorts, we wanted to determine the impact of the ocular surface condition on the accuracy of measurements of the anterior segment structures.

## Materials and methods

### Participants

This study was conducted following the tenants Declaration of Helsinki and approval from the Sandwell and West Birmingham NHS Trust Clinical Effectiveness and Safeguarding Group (Project Registration number #1843, and Project Registration date 08/10/2021). A data-sharing agreement was signed between Sandwell and West Birmingham NHS Trust and Karolinska Institutet. Informed consent was obtained from each patient. A total of 239 participants presenting to the inflammatory eye diseases clinic at the Birmingham and Midland Eye Centre (UK) underwent anterior segment imaging. Patients were categorized into six aetiological clinical groups. G1 = Sjögren’s syndrome, G2 = Immune-mediated OSD (Ocular Mucous Membrane Pemphigoid, Stevens-Johnson Syndrome / Toxic Epidermal Necrolysis, Graft-versus-Host Disease); G3 = Non-Immune-mediated OSD (Meibomian Glands Disease, Ocular Rosacea, Atopic Blepharo-keratoconjunctivitis); G4 = High Risk Corneal Transplantation Surgery; G5 = Miscellaneous (Neurotrophic, Injury/Trauma, Preservative Toxicity, Exposure keratopathy, Inherited); and G6 = Healthy controls (participants with no known ocular or systemic disorders and no previous ocular surgery). Only one eye per participant was included with at least 2 readable scans.

### Instrumentation and OCT measurements

The participants underwent nonmydriatic OCT imaging with the swept-source anterior segment Casia2 (Tomey, Japan). The Casia2 has a swept laser source of 1310 nm wavelength and performs up to 50,000 A-scans/second. The axial and transverse resolution are 10 μm and 30 μm, respectively. The maximum scan depth and width are approximately 13 mm and 16 mm, respectively. This instrument allows the possibility to image all the anterior segment of the eye including cornea, conjunctiva, anterior chamber, iris and both surfaces of the crystalline lens. In this study, the standard *anterior segment screening* mode was used which is composed of 16 radial scans delivered in approximately 0.3 s avoiding long and stressful ocular surface exposure. The repeated measurements were taken under repeatability conditions obtained with the same method, on identical test items, in the same laboratory, by the same operator, using the same equipment, and within short intervals of time (14, 15) by an experienced examiner (AR), with sufficient breaks in between to ensure good patient cooperation. Scans were repeated in there was poor fixation, lid blink during image capture, or if the scan was of unacceptable according to the instrument’s analysis software.

### Parameters analysed

**FIGURE 1:**
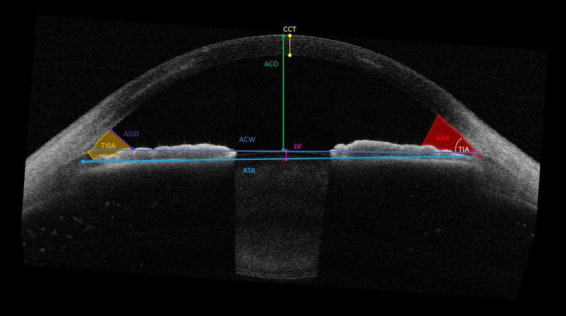
Parameters measured by the CASIA2 anterior chamber depth (ACD); the anterior chamber width (ACW), the angle opening distance (AOD) and angle recess area (ARA) 250 μm, 500 μm, and 750 μm, respectively; angle to angle (ATA); apical corneal thickness (CTApex) and thinnest corneal thickness (CTThin); Flat keratometry (Kf) and Steep keratometry (Ks); lens vault (LV); trabecular iris angle (TIA) and trabecular iris-space area (TISA) at 250 μm, 500 μm, and 750 μm, respectively.

A number of anterior segment measurements were analysed: (1) the ACD measured from the corneal endothelium level to the anterior lens surface, (2) the anterior chamber width (ACW) measured between the two scleral spurs, (3) the angle opening distance (AOD) between the posterior corneoscleral surface and the anterior iris surface perpendicular to the trabecular meshwork at 250 μm, 500 μm, and 750 μm from the scleral spur, respectively, (4) the angle recess area (ARA) formed by AOD, iris surface and the inner corneo-scleral wall traversed at the angle recess at 250 μm, 500 μm, and 750 μm, respectively, (5) angle to angle (ATA) measured between the angle recesses on the nasal and temporal sides, (6) apical corneal thickness (CTApex), (7) the thinnest corneal thickness (CTThin) measured between the anterior and posterior surfaces of the cornea on the apical point, (8) Anterior Flat keratometry (Kf) measured considering the flattest corneal radius in millimeters (mm), (9) Anterior Steep keratometry (Ks) measured considering the steepest corneal radius in millimeters (mm), (10) lens vault (LV) measured between the anterior crystalline lens surface and the horizontal line joining the two scleral spurs, (11) trabecular iris angle (TIA), and (12) trabecular iris-space area (TISA) measured between the apex in the iris recess and the scleral spur and the point on the iris perpendicularly at 250 μm, 500 μm, and 750 μm, respectively (Figure 1).

In addition, ocular surface metrics such as Ocular Surface Disease Index (OSDI^®^ questionnaire (OSDI) to assess the symptoms of dry eye disease and their impact on vision-related quality of life), fluorescein break-up time (FBUT) measured with 1% preservative-free fluorescein, tear meniscus height (TMH) at the lower eyelid margin, conjunctival inflammation (INFLCONJ) (16) and SICCA ocular staining score (OSS) (17) were measured, and the responses were also included in the analysis.

### Statistical analysis

The statistical analyses were performed using SPSS for Windows version 23.0 (SPSS Inc., Chicago, USA), and MATLAB (Mathworks inc., USA). The within-subject standard deviation (Sw), repeatability limits (Rlim) and coefficients of variation (CoVs) were used to describe the repeatability of each measurement parameter in each cohort. The Sw, which represents the repeatability of the measurements, was calculated with a one-way analysis of variance. The repeatability limit was calculated as [1.96⋅√2⋅*S*_*w*_], and it represents the expected limits that 95 % of the measurements should be within (18). The CoVs were calculated by dividing the Sw by the mean and then expressed in percentage. Data normality was tested using the Shapiro–Wilk test. The bivariate correlation analysis for non-normally distributed data was analyzed using the Spearman test. A guide to interpreting the correlation strength was derived from the recommendations of Navarro (19). A *p*-value of 0.05 was taken to be statistically significant.

## Results

**FIGURE 2:**
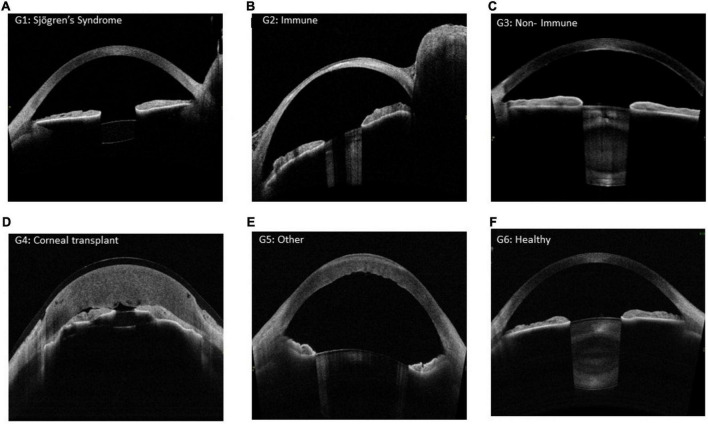
**(A)** Sjögren’s syndrome, **(B)** Immune, **(C)** Non- Immune, **(D)** Corneal Transplantation, **(E)** Other and **(F)** Healthy.

The participants characteristics are summarised in Table 1. The descriptive statistics of the analysed parameters and ocular surface metrics for each group are given in Table 2. Figure 2 shows example AS-OCT images from each group.

**TABLE 1:** Participants characteristics.

	All OSD Patients	Group 1 (Sjögren’s Syndrome)		Group 2 (Immune)		Group 3 (Non-Immune)		Group 4 (Corneal Transplantation)		Group 5 (Other)		Group 6 (Healthy)		*P*-Value
Biological sex (Male/Female)	239	4	46	28	32	31	29	13	4	4	8	13	27	< 0.001
Ethnicity*														
White	124	25		31		25		8		8		27		< 0.001
Mixed or multiple ethnic groups	5	2		1		1		0		0		1		0.639
Asian or Asian British	52	12		7		14		6		2		11		0.043
Black, Black British, Caribbean or African	12	5		1		4		1		0		1		< 0.001
Other ethnic group	5	0		0		5		0		0		0		< 0.001
Unknown group	41	6		20		11		2		2		0		< 0.001
	**[median (range)]**	**[median (range)]**		**[median (range)]**		**[median (range)]**		**[median (range)]**		**[median (range)]**		**[median (range)]**		***P*-Value**
Age (years)	[60 (17–96)]	[59 (27–88)]		[69 (17–89)]		[60 (20–96)]		[56 (30–91)]		[58 (42–96)]		[55 (35–96)]		0.123
OSDI TOTAL (score)	[35.40 (0.00–100.00)]	[52.50 (8.30–100)]		[18.80 (0.00–100)]		[33.30 (0.00–100)]		[55.20 (0–87.50)]		[44.40 (8.30–77.30)]		[18.53 (0.00–75)]		0.731
FBUT (s)	[5.00 (5.00–15.00)]	[4.00 (0.00–8.00)]		[5.00 (0.00–10.50)]		[4.50 (0.00–15.00)]		[5.00 (0.00–12.00)]		[4.50 (2.00–12.00]		[8.03 (2.61–12.52)]		0.289
TMH (mm)	[0.10 (0.00–0.43)]	[0.10 (0.00–0.30)]		[0.10 (0.00–0.30)]		[0.15 (0.00–0.40)]		[0.10 (0.00–0.30)]		[0.10 (0.10–0.30)]		[0.24 (0.06–0.43)]		0.541
INFLCONJ (score)	[0.00 (0.00–8.50)]	[0.00 (0.00–5.50)]		[0.00 (0.00–8.00)]		[0.00 (0.00–8.50)]		[0.00 (0.00–8.50)]		[0.00 (0.00–4.00]		[1.35 (0.01–4.18)]		0.111
OSS (score)	[2.00 (0.00–10.50)]	[4.00 (0.50–10–50)]		[2.00 (0.00–7.00)]		[2.25 (0.00–7.50)]		[2.75 (0.00–6.00)]		[2.50 (0.00–3.50)]		[1.52 (0.46–2.67)]		0.391

G1, Sjögren’s syndrome; G2, Immune; G3, Non-Immune; G4, Corneal Transplantation, G5 Other, Ocular Surface Disease [Neurotrophic (*n* = 4), Injury/Trauma (*n* = 3), Preservative Toxicity (*n* = 1), Exposure keratopathy (n = 3) and Inherited (*n* = 1)]; G6, Healthy controls; Clinical Features: OSDI, Ocular Surface Disease Index (patient symptomatology score); FBUT, Fluoresceine Break-up Time (patient tear film evaporation time in seconds); TMH, Tear Meniscus Height (patient tear film volume estimation in mm); INFLCONJ, Inflammation Conjunctival Score (patient conjunctival eye redness score); OSS, SICCA Ocular Staining Score; Data: reported as median and range (MIN-MAX, with p-values from Mann-Whitney U tests [*p* < 0.05; *p* < 0.01; *p* < 0.001; NS, Not Significant].

*As a matter of ease, groups were classified as follows: 1, White British | 2, White Irish | 3, White Gypsy or Irish Traveler | 4, Any other White background all under White; 5, Mix White and Black Caribbean | 6, Mix White and Black African | 7, Mix White and Asian | 8, Any Other Mixed/multiple ethnic background all under Mixed or multiple ethnic groups; 9, Indian | 10, Pakistani | 11, Bangladeshi | 12, Chinese | 13, Any other Asian background all under Asian or Asian British; 14, Black African | 15, Black Caribbean | 16, Any other Black/African/Caribbean background all under Black, Black British, Caribbean or African; 17, Arab | 18, Any other ethnic group all under Other ethnic group; rest of them as 19, Not known/not provided are all under unknown group.

**TABLE 2:** Summary of the anterior chamber parameters.

Groups Variables	Group 1 (Sjögren’s Syndrome)	Group 2 (Immune)	Group 3 (Non-Immune)	Group 4 (Corneal Transplant)	Group 5 (Other)	Group 6 (Healthy)
	**Mean ± STD**	**Mean ±** **STD**	**Mean ±** **STD**	**Mean ±** **STD**	**Mean ±** **STD**	**Mean ±** **STD**
**Anterior Chamber parameters**						
ACD	2.93 ± 0.46	3.02 ± 0.57	2.94 ± 0.45	3.54 ± 0.62	2.92 ± 0.43	3.08 ± 0.54
ACW	12.01 ± 0.51	11.97 ± 0.50	12.00 ± 0.45	12.03 ± 0.58	12.18 ± 0.67	11.96 ± 0.52
AOD250N	0.29 ± 0.13	0.29 ± 0.15	0.27 ± 0.11	0.39 ± 0.15	0.24 ± 0.09	0.27 ± 0.12
AOD250T	0.31 ± 0.16	0.31 ± 0.21	0.28 ± 0.14	0.36 ± 0.11	0.25 ± 0.13	0.32 ± 0.17
AOD500N	0.40 ± 0.20	0.40 ± 0.22	0.39 ± 0.17	0.63 ± 0.28	0.35 ± 0.13	0.41 ± 0.20
AOD500T	0.42 ± 0.22	0.44 ± 0.28	0.39 ± 0.20	0.55 ± 0.18	0.36 ± 0.21	0.47 ± 0.23
AOD750N	0.54 ± 0.27	0.57 ± 0.31	0.53 ± 0.25	0.91 ± 0.39	0.49 ± 0.22	0.59 ± 0.30
AOD750T	0.56 ± 0.28	0.62 ± 0.37	0.54 ± 0.28	0.78 ± 0.27	0.48 ± 0.25	0.64 ± 0.33
ARA250N	0.07 ± 0.04	0.06 ± 0.05	0.06 ± 0.03	0.09 ± 0.05	0.05 ± 0.02	0.06 ± 0.03
ARA250T	0.07 ± 0.04	0.08 ± 0.08	0.06 ± 0.04	0.08 ± 0.03	0.05 ± 0.03	0.07 ± 0.05
ARA500N	0.15 ± 0.08	0.15 ± 0.09	0.14 ± 0.06	0.22 ± 0.10	0.12 ± 0.05	0.14 ± 0.07
ARA500T	0.16 ± 0.09	0.17 ± 0.13	0.15 ± 0.08	0.19 ± 0.06	0.13 ± 0.07	0.17 ± 0.10
ARA750N	0.27 ± 0.14	0.27 ± 0.15	0.26 ± 0.11	0.41 ± 0.18	0.23 ± 0.09	0.27 ± 0.13
ARA750T	0.28 ± 0.15	0.31 ± 0.21	0.27 ± 0.13	0.36 ± 0.12	0.24 ± 0.12	0.31 ± 0.16
ATA	11.74 ± 0.54	11.66 ± 0.59	11.73 ± 0.43	11.78 ± 0.57	11.89 ± 0.64	11.72 ± 0.56
TIA250N	52.87 ± 17.08	53.81 ± 20.21	52.88 ± 15.59	63.75 ± 14.91	53.79 ± 17.41	50.68 ± 19.78
TIA250T	53.31 ± 18.84	53.99 ± 19.11	53.17 ± 18.92	60.69 ± 12.89	51.90 ± 21.86	53.74 ± 19.52
TIA500N	40.54 ± 14.22	41.30 ± 16.04	40.52 ± 12.54	53.92 ± 16.02	40.08 ± 13.31	40.86 ± 16.99
TIA500T	40.74 ± 15.78	42.71 ± 16.78	40.28 ± 16.05	50.29 ± 10.70	38.34 ± 16.23	44.01 ± 17.25
TIA750N	36.11 ± 13.67	37.83 ± 14.77	36.63 ± 12.49	51.04 ± 15.18	36.70 ± 13.21	38.31 ± 16.31
TIA750T	37.75 ± 13.86	39.78 ± 16.26	36.33 ± 14.70	47.87 ± 10.29	34.68 ± 13.66	40.55 ± 18.05
TISA250N	0.06 ± 0.03	0.06 ± 0.03	0.05 ± 0.02	0.08 ± 0.03	0.05 ± 0.02	0.05 ± 0.03
TISA250T	0.06 ± 0.03	0.07 ± 0.05	0.06 ± 0.03	0.07 ± 0.02	0.05 ± 0.02	0.06 ± 0.04
TISA500N	0.15 ± 0.07	0.14 ± 0.08	0.14 ± 0.06	0.21 ± 0.09	0.12 ± 0.05	0.14 ± 0.06
TISA500T	0.15 ± 0.08	0.16 ± 0.11	0.14 ± 0.07	0.19 ± 0.06	0.13 ± 0.06	0.16 ± 0.08
TISA750N	0.26 ± 0.13	0.27 ± 0.14	0.25 ± 0.11	0.40 ± 0.17	0.23 ± 0.09	0.27 ± 0.13
TISA750T	0.28 ± 0.14	0.29 ± 0.19	0.26 ± 0.12	0.35 ± 0.11	0.23 ± 0.12	0.30 ± 0.15
**Corneal parameters**						
CTApex	525.41 ± 41.73	529.70 ± 33.97	525.51 ± 48.69	520.62 ± 67.34	520.29 ± 52.15	522.46 ± 38.84
CTThin	513.60 ± 52.10	516.86 ± 39.26	504.04 ± 56.71	471.59 ± 83.17	498.08 ± 92.09	514.31 ± 37.43
Kf	48.24 ± 2.08	48.96 ± 2.59	48.88 ± 3.81	49.54 ± 5.21	47.29 ± 3.05	47.91 ± 1.93
Ks	49.79 ± 2.70	50.26 ± 3.31	50.22 ± 4.14	52.69 ± 6.27	48.46 ± 2.51	49.06 ± 2.01
LV	0.26 ± 0.41	0.15 ± 0.51	0.24 ± 0.38	−0.22 ± 0.48	0.32 ± 0.32	0.12 ± 0.51

Parameters included as mean ± STD: anterior chamber depth (ACD), anterior chamber width (ACW), angle opening distance (AOD), angle recess area (ARA) 250 μm, 500 μm, and 750 μm, angle to angle (ATA), trabecular iris angle (TIA), trabecular iris-space area (TISA) at 250 μm, 500 μm, and 750 μm, apical corneal thickness (CTApex), thinnest corneal thickness (CTThin); Flat keratometry (Kf) and Steep keratometry (Ks) and lens vault (LV).

### Anterior chamber parameters

**FIGURE 3:**
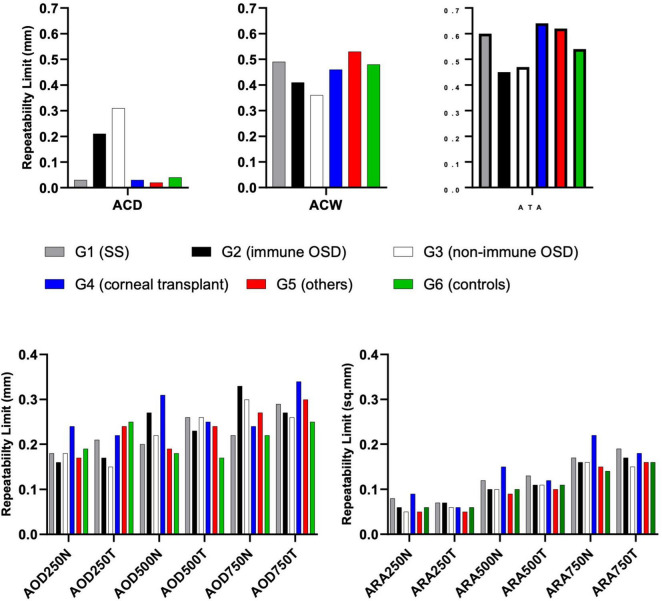
Repeatability limits for ACD, ACW, ATA, AOD and ARA at 250, 500 and 750 μm, respectively. Refer to Table 2 for abbreviations.

Figure 3 shows the Rlim values for ACD (upper left panel), ACW (upper central panel) and ATA (upper right panel). The respective values for ACD in immune and non-immune OSD cohorts were higher compared to other cohorts whereas the trend was opposite for the ACW and ATA measurements. The coefficients of variations were similar amongst all three metrics (Table 3). Most of the AOD (bottom right) and ARA (bottom left) RLim measured at 250 μm, 500 μm, and 750 μm were higher in the corneal transplant group (G4) (Figure 3). However, most of the AOD and ARA CoVs were slightly higher in the Neurotrophic, Injury/Trauma, Preservative Toxicity, Exposure keratopathy and Inherited (G5) compared to other groups except for most of the ARA values that were similar to the SS group (G1) Table 3. There were no significant differences in the Sw among the groups for any of these parameters (*p* > 0.05).

**TABLE 3:** Coefficient of variation (%) for ACD, ACW, ATA, AOD and ARA at 250, 500 and 750 μm, respectively.

Groups Variables	G1 (Sjögren’s Syndrome)	G2 (Immune)	G3 (Non-Immune)	G4 (Corneal Transplant)	G5 (Other)	G6 (Healthy)
**Coefficients of Variation (%)**						
ACD	0.4	2.5	3.8	0.3	0.2	0.5
ACW	1.5	1.2	1.1	1.4	1.6	1.4
AOD250N	22.4	20.1	24.3	22.5	26.2	24.6
AOD250T	25.0	19.9	19.5	22.0	34.2	27.9
AOD500N	17.7	23.6	20.4	17.8	20.3	16.1
AOD500T	22.7	18.5	23.5	16.8	24.2	13.5
AOD750N	14.9	20.9	20.4	9.5	20.0	13.4
AOD750T	18.4	15.7	17.2	15.8	22.0	14.1
ARA250N	41.9	31.6	33.5	36.8	37.9	37.4
ARA250T	36.3	31.0	32.4	28.1	34.2	30.4
ARA500N	28.1	23.9	25.7	25.7	26.4	24.7
ARA500T	28.4	23.4	26.3	21.9	28.5	22.8
ARA750N	22.4	21.6	22.5	18.9	23.2	18.7
ARA750T	24.4	20.0	20.1	17.6	25.1	18.9
ATA	1.9	1.4	1.5	2.0	1.9	1.7

Refer to Table 2 for abbreviations.

### Corneal parameters

**FIGURE 4:**
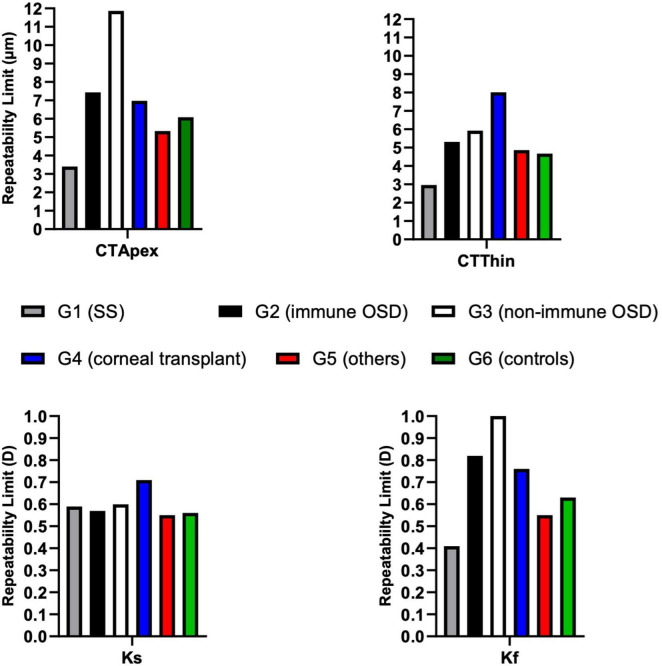
Repeatability limits for CTApex, CTThin, Kf and Ks. Refer to Table 2 for abbreviations.

RLim for CTApex and Kf were higher in non-immune OSD cohort while CTThin and Ks showed higher RLim and CoVs in the corneal transplant group (G4) (Figure 4 and Table 4). There were no significant differences in the Sw among the groups for any of these parameters (*p* > 0.05).

**TABLE 4:** Coefficients of variation (%) for corneal thickness and curvature.

Groups Variables	G1 (Sjögren’s Syndrome)	G2 (Immune)	G3 (Non-Immune)	G4 (Corneal Transplant)	G5 (Other)	G6 (Healthy)
**Coefficients of Variation (%)**						
CTApex	0.2	0.5	0.8	0.5	0.4	0.4
CTThin	0.2	0.4	0.4	0.6	0.4	0.3
Kf	0.3	0.6	0.7	0.6	0.4	0.5
Ks	0.4	0.4	0.4	0.5	0.4	0.4

Refer to Table 2 for abbreviations.

### Other anterior segment parameters

**FIGURE 5:**
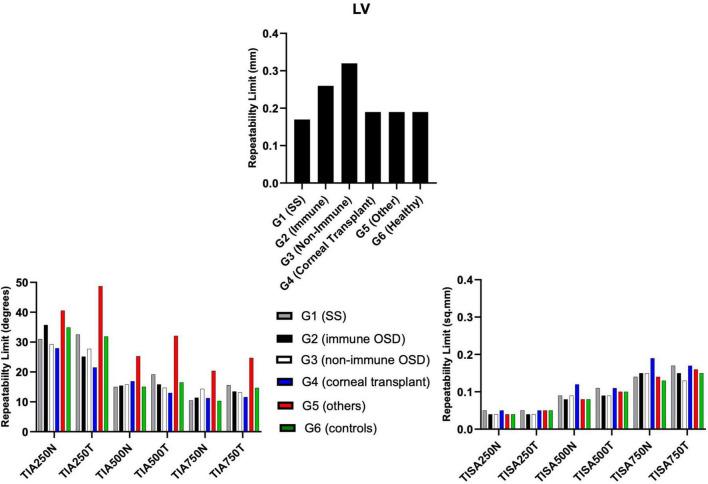
Repeatability limits for LV, TIA and TISA at 250 μm, 500 μm, and 750 μm, respectively. Refer to Table 2 for abbreviations.

All TIA RLim measured at 250 μm, 500 μm, and 750 μm were higher in the G5 cohort, with a similar trend observed for the CoVs values. Most of the TISA RLim were higher in the G4 cohort while for the CoVs highest values were seen in G5 (Figure 5). CoVs value for LV was majorly higher in the G2 cohort (Table 5). There were no significant differences in the Sw among the groups for any of these parameters (*p* > 0.05).

**TABLE 5:** Coefficients of variation (%) for LV, TIA and TISA at 250 μm, 500 μm, and 750 μm, respectively.

Groups Variables	G1 (Sjögren’s Syndrome)	G2 (Immune)	G3 (Non-Immune)	G4 (Corneal Transplant)	G5 (Other)	G6 (Healthy)
**Coefficients of Variation (%)**						
LV	23.3	63.5	48.1	31.4	21.2	58.2
TIA250N	21.2	23.9	20.0	15.8	27.2	24.8
TIA250T	22.0	16.8	18.8	12.8	33.9	21.4
TIA500N	13.4	13.5	14.1	11.3	22.8	13.3
TIA500T	17.0	13.4	13.2	9.3	30.2	13.5
TIA750N	10.5	10.9	14.1	8.0	20.0	9.7
TIA750T	14.9	12.2	13.1	8.8	25.7	13.1
TISA250N	28.5	25.7	29.3	26.3	31.9	29.6
TISA250T	28.2	24.1	24.2	23.8	33.9	28.7
TISA500N	22.3	21.5	24.0	21.6	24.0	21.8
TISA500T	25.3	20.4	23.6	20.6	28.4	21.7
TISA750N	19.1	20.3	21.6	16.8	21.8	17.0
TISA750T	22.7	18.4	18.2	17.0	25.0	18.2

Refer to Table 2 for abbreviations.

### Discussion

This is the first study assessing the repeatability of an anterior segment OCT in different ocular surface disorder groups for measuring various anterior segment parameters. Our results show that the repeatability for the anterior chamber and corneal parameters were similar among all groups and the presence of ocular surface disease did not impact the accuracy. Similarly, the RLim for the LV and TISA were also similar among all groups.

Ocular surface inflammation is a pivotal driver of dry eye for patients with underlying immune-mediated and conditions and those without (20). Additionally, aging can negatively impact on ocular surface health both in the ‘healthy’ population and those known to ocular surface disorders.

There is a wide range of new techniques that are able to provide quantitative parameters of different anterior segment structures. Although, the damaging effect of ocular surface disorders might have an impact on the performance of these innovative tools. In fact, the ocular surface is the first media that these devices encounter during the measurements, and hence its homeostasis plays a crucial role in the precision of the quantitative metrics (21). Nevertheless, in this study we have not considered ocular surface metrics such as corneal scarring, vascularisation and the presence of conjunctival scarring that appear in severe ocular surface disease (22). The availability of anterior segment imaging devices in public hospitals is still limited due to their cost, maintenance and measurement efficacy (need of repeated measurements/time allocated for each consultation) (23). The use of OCT in the anterior segment is constantly increasing with newer devices able to provide faster scanning modes and images with higher resolution (∼15 μm). More recently, ss-AS-OCT has proven to be the foremost technique in detailing the front structures of the eye. Newer AS-OCTs can provide better scanning with deeper tissue penetration and enhanced scanning modes to depict anterior segment pathologies (24). Also, they appeared to be reasonably repeatable showing excellent intradevice measurement in healthy cohorts (25). However, as shown in a previous study considering posterior segment OCT, it appears that dry eye can be responsible for the reduction in scan quality compromising repeatability (26).

In the present study, the RLim values for the ACD measurements were larger in both ocular surface disease cohorts (G2: Immune and G3: Non-immune) compared to the other groups, but the differences were not significantly different (*p* > 0.05). It is shown that severe and chronic ocular surface disease patients have altered endothelial cell layer caused by the reduced corneal nerves density (27). Also, Belmonte et al. (28) suggested that prolonged inflammation stress and reduced tear film availability over the ocular surface might lead to abnormal corneal nerves ending developing further symptomatology and affecting the deeper corneal structure. This could result in decreased precision of the endothelial layer segmentation. The RLim for ACW measurements were on a similar magnitude among the groups, and the CoVs never exceeded 1.6%. The ACW is a measure of the distance between the scleral spurs, hence altercations of the ocular surface may not affect the precision of this parameter. Although, chronic dryness might affect the scleral thickness due to the tissue’s perpetual inflammation (29), it might not influence the precision of the ACW measurement. Similar to the ACW, the RLim for ATA was similar among the groups and the CoVs never exceeded 2%. Repeatability of AOD and ARA, there is no clear trend among the groups. However, the RLim for G5 showed larger values at many points but this could also be attributed to smaller sample size in that group. As Pentacam devices are still one of the most considered instruments in public hospital settings in the UK, we decided to compare the corneal thickness measurements in our cohorts with similar studies done with them but acknowledging these might be not the gold standard for pachymetry measurements.

Corneal thickness measurements variation was seen among the RLim values for the different clinical groups. However, the CoVs were less than 1%. Previous studies using Pentacam show contradicting results. Lee et al. reported that the repeatability with Pentacam for the central corneal thickness measurement was worse for the dry eye group (34 subjects) that predominantly had aqueous-deficient dry eye compared to controls (30). However, another study that included dry eyes subjects with causes similar to that included in the present study showed that the repeatability was similar between dry eye (138 subjects) and control groups (13). Not surprisingly, the anterior corneal power (the RLim for the Kf) was different amongst groups. Whereas when evaluating Ks, the RLim was more similar amongst the groups. However, the CoV values of Kf and Ks were lower than 1 % for all groups confirming that these measurements were not affected by the conditions of the ocular surface. Corneal topographic measurements were shown to be highly repeatable in severe dry eye disease using Pentacam (13). Another study using the IOLMaster 500 evaluated the influence of artificial tears on the keratometric measurements in cataract patients. The repeatability was found to be similar between the control and dry eye groups before instillation of artificial tears. However, the variation in the measurements was larger in the dry eye group after instillation of artificial tears (13, 31). Both previous studies mentioned, and our present results suggest that the repeatability of the keratometer does not vary largely among the dry eye groups. This could be related to the fast acquisition time.

The RLim for LV showed similar trend to that of ACD, with G2 and G3 showing larger values. Regarding TIA and TISA, G5 showed the largest CoV values, which could be related to the low sample size in this group.

Previous swept-source AS-OCT studies have also reported that the repeatability of anterior chamber angle parameters is lower compared to other anterior segment parameters in healthy eyes (32, 33). In recent years, TIA and TISA values have been widely accepted as objective metrics in determining angle opening in normal and disease populations (34, 35). If these metrics are meant to be used in the clinic, the measurement variability should be taken into consideration. The repeatability values presented in this study are based on measurements using the anterior segment screening protocol, which contains 16 radial B-scans and takes 0.3 s. A previously studied by Liu et al. (36) using the same instrument, the authors reported better repeatability values for the anterior chamber angle parameters compared to the present study but without considering ocular surface disease cohorts. However, in that study the measurements were performed with the anterior chamber angle scan protocol, which contains 128 radial B-scans and takes 2.3 seconds. It should be considered that dry eye subjects might have trouble keeping their eyes open and fixate if the acquisition time is longer.

Correlations results between dry eye metrics and AS-OCT measurements have been shown to be controversial too: in a research published by Diana and Ana (37) none of the dry eye metrics such as tear film thickness, tear meniscus area and tear meniscus height assessed via AS-OCT were discriminative between healthy and disease cohorts. However, findings from Schmidl et al. (38) showed that tear film thickness values measured with AS-OCT with dry eye correlated with patient’s symptomatology. this discrepancy could be due to the fact that dry eye disease is a multifactorial disease: (3) and many other variables can also play a role in the diagnosis and severity of the condition across different patients.

This is the first study where ss-AS-OCT was used in OSD cohorts that included severe immune-mediated conditions such as Sjögren’s Syndrome, Ocular Mucous Membrane Pemphigoid, Stevens Johnson-Syndrome, etc. Our results show that Tomey CASIA 2 demonstrates good repeatability when dealing with OSD patients.

The results of the present study show good repeatability for measuring anterior segment parameters measured with Tomey CASIA 2 in OSD cohorts. These results demonstrate that the clinical measurement protocol used in healthy controls could be also used in OSD subjects.

## Data availability statement

The raw data supporting the conclusions of this article will be made available by the authors, without undue reservation.

## Ethics statement

This study was conducted following the tenants Declaration of Helsinki and approval from the Sandwell and West Birmingham NHS Trust Clinical Effectiveness and Safeguarding Group (Project Registration #1843). The studies were conducted in accordance with the local legislation and institutional requirements. The participants provided their written informed consent to participate in this study.

## Author contributions

AR: Conceptualization, Data curation, Investigation, Methodology, Validation, Writing−original draft, Writing−review and editing, Funding acquisition. AV: Conceptualization, Formal analysis, Investigation, Methodology, Validation, Writing−original draft, Writing−review and editing. SR: Conceptualization, Data curation, Funding acquisition, Investigation, Project administration, Validation, Writing−review and editing. AD-V: Conceptualization, Formal analysis, Investigation, Supervision, Writing−original draft, Writing−review and editing, Methodology, Validation.
